# Glymphatic Dysfunction in Neuro-Pulmonary Complications Following Subarachnoid Hemorrhage: A New Perspective on Brain–Lung Axis Disruption

**DOI:** 10.3390/cells14211739

**Published:** 2025-11-05

**Authors:** Eun Chae Lee, Jae Sang Oh

**Affiliations:** 1 Medical Life Science, College of Medicine, The Catholic University of Korea, Seoul 06591, Republic of Korea; lec9589@gmail.com; 2 Department of Neurosurgery, Uijeongbu St. Mary’s Hospital, College of Medicine, The Catholic University of Korea, Seoul 06591, Republic of Korea

**Keywords:** glymphatic dysfunction, subarachnoid hemorrhage, brain–lung axis, lymphatic system, aquaporin-4, central nervous system, damage-associated molecular patterns (DAMPs)

## Abstract

Subarachnoid hemorrhage (SAH), often resulting from aneurysmal rupture, remains a life-threatening cerebrovascular disorder with high morbidity and mortality. While previous research has focused primarily on cerebral damage and neurological outcomes, growing evidence suggests that SAH also causes systemic complications, including pulmonary dysfunction. The underlying mechanisms linking SAH to lung injury, however, are not fully understood. The glymphatic system, a perivascular network that facilitates the clearance of cerebrospinal fluid (CSF) and interstitial waste from the brain, plays a critical role in maintaining central nervous system (CNS) homeostasis. Aquaporin-4 (AQP4) water channels, predominantly expressed in astrocytic end feet, are essential for efficient glymphatic flow. Emerging studies have shown that SAH impairs glymphatic function by disrupting AQP4 polarity and CSF circulation, resulting in the accumulation of neurotoxic substances and neuroinflammation. Recent findings further suggest that glymphatic dysfunction may exert systemic effects beyond the CNS, contributing to a breakdown of the brain–lung axis. The release of pro-inflammatory cytokines, blood degradation products, and damage-associated molecular patterns (DAMPs) into systemic circulation can promote pulmonary endothelial injury and trigger immune responses in the lungs. This phenomenon is exacerbated by impaired clearance via the glymphatic system, amplifying systemic inflammation and increasing the risk of acute lung injury (ALI) or neurogenic pulmonary edema (NPE). This review proposes a novel perspective linking glymphatic impairment with pulmonary complications after SAH. Understanding this connection could open new therapeutic avenues—such as targeting AQP4 function, enhancing CSF circulation, or modulating the inflammatory response—to mitigate both neurological and respiratory sequelae in SAH patients.

## 1. Introduction

Subarachnoid hemorrhage (SAH) is a catastrophic neurological event, most caused by the rupture of intracranial aneurysms, with an annual incidence of approximately 9 per 100,000 person-years worldwide [1,2,3]. Although it accounts for only 5% of all strokes, SAH disproportionately contributes to stroke-related mortality and long-term disability, particularly among younger individuals [4,5,6]. Despite recent advancements in early surgical clipping or endovascular coiling, complications such as delayed cerebral ischemia (DCI), neuroinflammation, and systemic organ dysfunction remain major obstacles to recovery [4,7,8,9,10].

Traditionally, SAH has been examined primarily through a neurocentral lens, emphasizing cerebral vasospasm, hydrocephalus, and blood–brain barrier (BBB) disruption as central mechanisms of secondary injury [11,12,13]. However, the growing body of literature suggests that SAH triggers multi-organ dysfunction, particularly involving the cardiopulmonary system [14,15]. Among these, pulmonary complications are both common and clinically significant. For instance, a multicenter study involving SAH patients reported that 31% developed acute lung injury (ALI), which was independently associated with increased ICU mortality and prolonged mechanical dependence [16,17]. Conditions such as neurogenic pulmonary edema (NPE), ALI, and acute respiratory distress syndrome (ARDS) often emerge within 48–72 h post-hemorrhage and are linked to elevated systemic inflammation, catecholamine surges, and impaired autonomic regulation [18,19].

**Figure 1:**
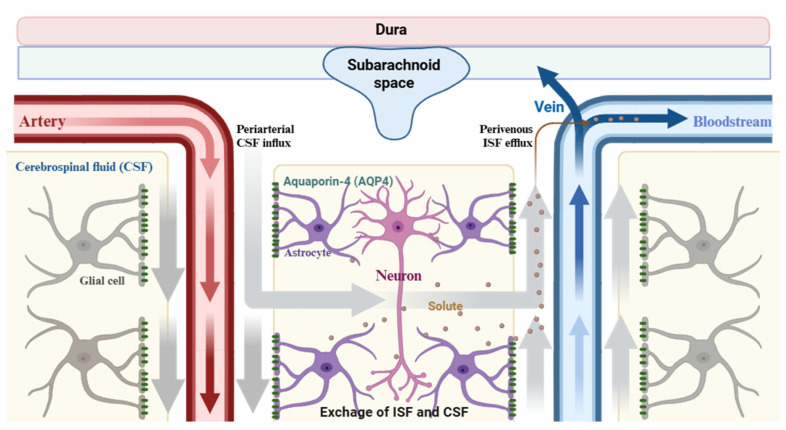
Schematic illustration of the glymphatic system and cerebrospinal fluid (CSF) dynamics in the brain. CSF flows from the subarachnoid space along periarterial spaces into the brain parenchyma. This influx is facilitated by aquaporin-4 (AQP4) water channels expressed on astrocytic end feet. Within the interstitial space, CSF mixes with interstitial fluid (ISF), enabling the clearance of solutes and metabolic waste from the brain. The mixed fluid then drains along perivenous pathways into the venous circulation. Neurons, astrocytes, and glial cells are involved in the exchange and regulation of CSF and ISF, which is critical for maintaining brain homeostasis. Disruption of this system is implicated in various neurological disorders.

One of the emerging players in post-SAH pathophysiology is the glymphatic system—a recently discovered perivascular network responsible for the clearance of metabolic waste, interstitial solutes, and neurotoxic proteins from the central nervous system [20,21]. The glymphatic system is a recently discovered waste clearance pathway in the brain, driven by cerebrospinal fluid (CSF) movement along perivascular spaces and facilitated by aquaporin-4 (AQP4) water channels on astrocytic end-feet [22,23]. It plays a critical role in removing metabolic byproducts such as amyloid-β and tau, and has been implicated in neurodegenerative disorders like Alzheimer’s disease. Understanding this system is essential not only for neurologists and neurosurgeons but also for clinicians and researchers in other disciplines [23]. The system is driven by cerebrospinal fluid (CSF) influx along para-arterial spaces, facilitated by the astrocytic water channel aquaporin-4 (AQP4), and efflux via perivenous routes into meningeal lymphatics [20,24,25]. Under physiological conditions, glymphatic transport is particularly active during sleep and contributes to central nervous system homeostasis by eliminating amyloid-β, tau, and inflammatory mediators [26,27] (Figure 1).

However, in the setting of SAH, glymphatic flow becomes severely impaired due to increased intracranial pressure (ICP), redistribution of AQP4 from astrocytic end feet, and inflammatory astrogliosis [28,29]. Experimental models show that SAH significantly reduces CSF influx and tracer clearance in glymphatic pathways, leading to the accumulation of damage-associated molecular patterns (DAMPs), oxidative stress, and neuroinflammation [30,31]. Recent research has indicated that glymphatic dysfunction may act as an early event triggering neuroinflammation and contributing to adverse neurological outcomes in SAH [32].

**Figure 2:**
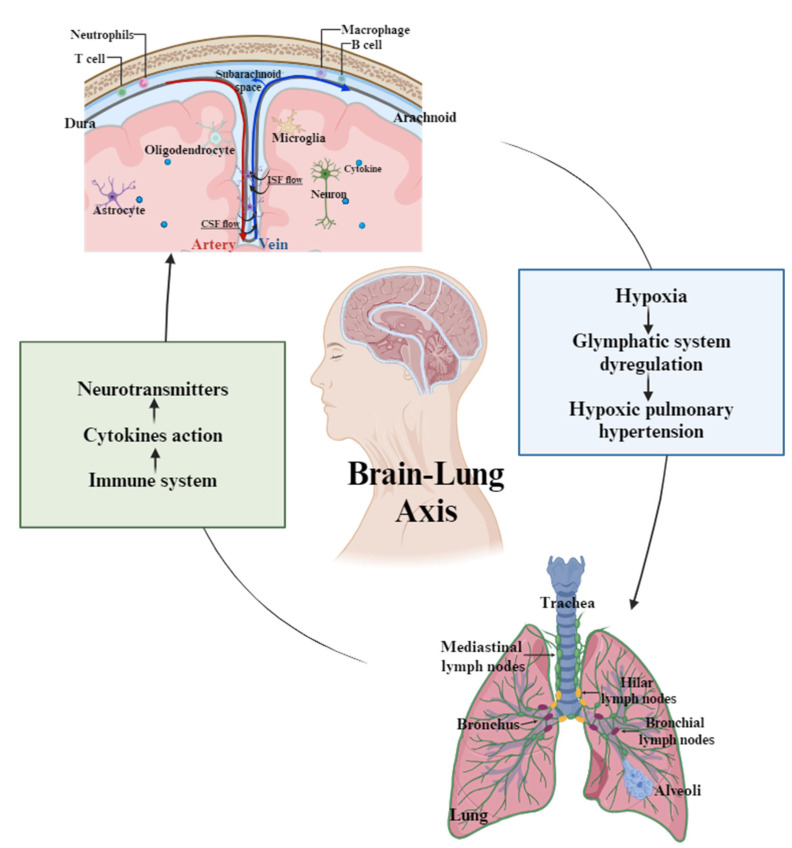
Representative mechanisms underlying the interaction between the glymphatic and lymphatic systems in the brain–lung axis. The figure illustrates the bidirectional communication between the brain and lungs, highlighting the roles of glymphatic circulation and neuroimmune signaling. In the brain, cerebrospinal fluid (CSF) flows along perivascular spaces and exchanges with interstitial fluid (ISF), facilitating the clearance of metabolic waste. This process involves various glial cells, including astrocytes, oligodendrocytes, and microglia. Cytokines and other immune mediators modulate neuronal activity and glymphatic function. Pulmonary hypoxia can disrupt glymphatic circulation, contributing to the development of hypoxic pulmonary hypertension. Conversely, immune responses originating in the lungs—such as cytokine release—can influence brain homeostasis via systemic immune signaling and neurotransmitter modulation. This reciprocal interaction represents a critical pathway in the pathophysiology of both neurological and pulmonary disorders.

Under hypoxic pulmonary conditions, we observed significant alterations in glymphatic function, including reduced CSF–ISF exchange and decreased expression of astrocytic AQP4 channels, indicating impaired waste clearance [33]. This dysfunction may contribute to the development of hypoxia-induced pulmonary hypertension. Conversely, activation of pulmonary immune responses led to increased systemic cytokine levels, which corresponded with changes in neuroinflammatory markers and altered neuronal activity in the cortex and hippocampus. These findings suggest that immune signals originating in the lungs can influence brain homeostasis through peripheral immune-to-brain communication pathways [34,35,36] (Figure 2).

Together, these results underscore a dynamic, reciprocal interaction between pulmonary and cerebral systems, with implications for understanding the pathophysiology of both neurological and respiratory disorders.

More importantly, recent data suggest that impaired glymphatic and meningeal lymphatic drainage may also have systemic implications, particularly in the context of the brain–lung axis [31,37,38]. Blood degradation products and pro-inflammatory cytokines released after SAH, such as IL-6, TNF-α, and HMGB1, can enter systemic circulation due to compromised CSF clearance, leading to pulmonary endothelial damage and immune cell infiltration [39,40,41]. Experimental evidence suggests that SAH induces pulmonary complications such as increased vascular permeability and immune cell activation, which can be alleviated by promoting glymphatic function via low-frequency CSF pulsation [42].

Moreover, the discovery of meningeal lymphatic vessels has revolutionized our understanding of CNS–immune communication. These vessels, which drain into cervical lymph nodes, serve as a critical bridge between the CNS and peripheral immune system. Impaired lymphatic drainage following SAH could hinder antigen clearance and amplify systemic immune dysregulation, further contributing to lung injury [43,44]. This bidirectional communication between brain and lung where neural injury exacerbates respiratory dysfunction and vice versa highlights the clinical importance of maintaining both neurological and systemic homeostasis.

Given this complex and interconnected pathophysiology, there is an urgent need to reframe our approach to SAH from a single-organ disorder to a multi-system syndrome. This review aims to synthesize current knowledge on glymphatic dysfunction and pulmonary complications following SAH, emphasizing the glymphatic–lymphatic axis as a potential mechanistic link. By exploring novel therapeutic strategies such as targeting AQP4 expression, modulating meningeal lymphatics, or attenuating systemic inflammation, we may better address both neurological and respiratory sequelae of SAH and ultimately improve patient outcomes.

This review aims to (1) summarize the pathophysiology of glymphatic dysfunction following subarachnoid hemorrhage (SAH), (2) synthesize evidence from animal and human studies, (3) describe the brain–lung axis and the mechanisms of pulmonary complications, and (4) discuss potential therapeutic approaches and future research directions.

## 2. Current Evidence and Pathophysiology

### 2.1. An Evolving Understanding of Glymphatic Biology

Since its initial characterization in 2012, the glymphatic system has revolutionized our understanding of how the brain clears metabolic waste and maintains interstitial homeostasis [20,45]. Unlike peripheral organs, the brain lacks classical lymphatic vessels within its parenchyma. Instead, it relies on a unique perivascular network that facilitates the convective flow of cerebrospinal fluid (CSF) into the brain interstitial and the clearance of solutes via perivenous pathways [22]. This system, termed the “glymphatic” pathway—so named for its dependence on glial cells and lymphatic-like function—is critically regulated by the water channel aquaporin-4 (AQP4), which is densely localized on astrocytic end feet [24].

In physiological states, glymphatic activity is most robust during sleep, particularly in the non-REM phase, when neuronal activity diminishes and interstitial space expands by up to 60%, promoting CSF influx and solute clearance [26,46]. This mechanism is believed to contribute significantly to the removal of neurotoxic proteins such as amyloid-β and tau, with implications for neurodegenerative diseases including Alzheimer’s and Parkinson’s disease.

However, the system is highly sensitive to pathological perturbations. After subarachnoid hemorrhage (SAH), several converging factors impair glymphatic function:

1. Elevated intracranial pressure (ICP) reduces the pressure gradient driving CSF influx into periarterial spaces [47];
2. Depolarization of AQP4 channels due to reactive astrogliosis disrupts the directional water flow necessary for convective exchange [42];
3. Obstruction of perivascular spaces by blood components and cellular debris hinders CSF movement and promotes local inflammation [48].

In parallel, researchers have begun to investigate how systemic factors including cardiovascular health, respiration, and immune status modulate glymphatic flow. Arterial pulpability, which provides a mechanical driving force for CSF influx, is diminished during systemic hypotension or vascular dysregulation, both of which are common following SAH [49]. Similarly, respiration-linked oscillations contribute to venous outflow and CSF clearance, implying that respiratory compromise (as seen in SAH-related pulmonary dysfunction) could indirectly impair glymphatic efficiency.

Recent advances in imaging, including dynamic contrast-enhanced MRI and two-photon microscopy, have enabled in vivo visualization of glymphatic flow, allowing researchers to quantify impairment in various disease states [50]. These tools have confirmed that glymphatic dysfunction precedes and predicts neuroinflammatory cascades, offering both diagnostic and therapeutic insights.

Importantly, the discovery of meningeal lymphatic vessels, which provide a physical route for glymphatic efflux to cervical lymph nodes, has added a critical dimension to this evolving model [51]. These vessels are now seen as essential partners in the brain’s waste clearance and immune surveillance. Dysfunction in this complementary system further exacerbates the accumulation of CNS waste and amplifies neuroinflammation [52].

In summary, glymphatic biology is undergoing a paradigm shift from a localized brain-cleansing mechanism to a dynamic, systemically influenced network with implications for neuroimmune regulation, inter-organ communication, and disease propagation. In the context of SAH, understanding this evolving system offers a framework for rethinking how primary brain injury leads to secondary systemic dysfunction and opens avenues for integrated therapeutic approaches.

### 2.2. Glymphatic Dysfunction Following SAH

Subarachnoid hemorrhage (SAH) induces a cascade of pathological changes that critically impair the function of the glymphatic system [44]. One of the most immediate consequences of SAH is a sudden and dramatic elevation in intracranial pressure (ICP), which compresses the perivascular spaces essential for CSF influx [53]. This mechanical disruption collapses the arterial perivascular channels through which CSF normally enters the brain parenchyma, thereby halting the convective bulk flow that drives glymphatic clearance.

The presence of extravasated blood in the subarachnoid space further compounds the dysfunction [54]. Hemolysis releases free hemoglobin, heme, and iron—all of which are potent pro-oxidants that catalyze lipid peroxidation and amplify reactive oxygen species (ROS) generation [55,56]. Thrombin and fibrinogen, released during coagulation, also exert pro-inflammatory and neurotoxic effects, promoting endothelial activation and astrocytic swelling [57]. These blood-derived components not only obstruct the perivascular routes directly but also trigger astrocyte reactivity (astrogliosis).

Astrocytes, which play a central role in glymphatic flow regulation through their expression of aquaporin-4 (AQP4) water channels, undergo morphological and functional changes in response to injury [58]. Reactive astrocytes exhibit a redistribution of AQP4 from their perivascular end feet to nonspecific cellular regions, a phenomenon known as AQP4 depolarization [59]. This spatial disorganization severely impairs the directional water transport required for effective CSF–interstitial fluid exchange [60]. Animal models of SAH and traumatic brain injury have shown that AQP4 misvocalization correlates with impaired glymphatic clearance, increased interstitial solute accumulation, and worsened neuroinflammation [24,60].

Moreover, SAH-induced blood–brain barrier (BBB) disruption exacerbates glymphatic failure by allowing peripheral immune cells and plasma proteins to infiltrate the CNS. This breach intensifies local cytokine release (e.g., IL-1β, TNF-α), creating a positive feedback loop of inflammation that impairs vascular compliance and reduces arterial pulpability both key drivers of glymphatic inflow [61,62]. Studies using real-time imaging have shown that reduced pulpability post-SAH leads to markedly decreased CSF tracer movement along the para-arterial spaces [63].

The cumulative effect of these alterations is a toxic neuro-environment characterized by the buildup of interstitial metabolites such as amyloid-β, tau, and lactate, which further impair neuronal function and synaptic integrity [64,65]. Importantly, impaired glymphatic clearance is now being linked to chronic neurodegenerative processes following SAH, suggesting that acute injury to this system may have long-lasting consequences for brain health [66].

In sum, SAH impairs glymphatic function via mechanical, cellular, and molecular mechanisms, disrupting the delicate balance of CSF circulation, immune signaling, and waste clearance. These changes not only potentiate secondary brain injury but may also initiate systemic immune responses with consequences that extend beyond the CNS.

### 2.3. Pulmonary Complications Linked to Glymphatic Impairment

The interplay between brain injury and pulmonary dysfunction has long been observed in clinical settings, with subarachnoid hemorrhage (SAH) often precipitating a range of acute lung complications, including neurogenic pulmonary edema (NPE), acute lung injury (ALI), and in severe cases, acute respiratory distress syndrome (ARDS) [67,68]. While the neural and neurohumoral pathways contributing to these complications have been partially elucidated, such as catecholamine surges, sympathetic overactivation, and systemic inflammatory response, emerging evidence suggests that glymphatic dysfunction and impaired brain waste clearance may be a central mediator in this brain–lung axis [69,70].

ARDS is defined according to the Berlin criteria as acute onset hypoxemia with bilateral pulmonary infiltrates not fully explained by cardiac failure or fluid overload [71,72]. ALI represents a less severe manifestation with similar pathophysiology but less severe hypoxemia. Neurogenic Pulmonary Edema (NPE) refers to acute, non-cardiogenic pulmonary edema resulting from massive sympathetic discharge after acute brain injury, leading to increased pulmonary vascular permeability. Following SAH, the impaired clearance of metabolic byproducts, DAMPs, and inflammatory cytokines due to glymphatic failure creates a pro-inflammatory intracranial milieu [73,74]. These molecules such as HMGB1, IL-6, and TNF-α can leak into the systemic circulation either through a compromised blood–brain barrier or via meningeal lymphatic vessels, which drain into the deep cervical lymph nodes and eventually into systemic lymphatic and venous systems [37,75,76,77]. This pathway represents a direct immunologic and molecular communication route between the brain and peripheral organs.

Once in systemic circulation, these mediators act on distant organs, with the lungs being particularly susceptible due to their extensive capillary network and high immune surveillance. Circulating cytokines and DAMPs interact with alveolar macrophages, pulmonary endothelial cells, and neutrophils, promoting the release of further pro-inflammatory mediators, increased vascular permeability, and neutrophil extracellular trap (NET) formation [78,79]. This cascade results in alveolar flooding, interstitial edema, and impaired gas exchange—hallmark features of neurogenic pulmonary edema and ARDS.

Recent animal studies have supported this hypothesis by showing that experimental SAH leads to marked pulmonary inflammation, histologic evidence of alveolar damage, and increased lung wet-to-dry weight ratios [80]. Notably, these effects are attenuated in models where glymphatic outflow is preserved or enhanced, such as via AQP4 stabilization or pharmacologic modulation of CSF dynamics.

In addition, the impaired clearance of CNS antigens due to glymphatic dysfunction may also contribute to immune priming and autoimmunity. Meningeal lymphatic vessels normally shuttle CNS-derived antigens to peripheral lymph nodes, where immune tolerance is maintained [81,82]. When this drainage is compromised, abnormal antigen presentation may lead to exaggerated systemic immune responses or even loss of self-tolerance, potentially exacerbating pulmonary immune injury.

An additional factor is systemic hypoxia resulting from pulmonary complications, which can further impair glymphatic clearance in a vicious cycle. Hypoxia reduces arterial pulpability and lowers intracranial compliance, both of which are essential for maintaining glymphatic flow [83]. Thus, pulmonary dysfunction both results from and contributes to worsening brain pathology, highlighting the bidirectional nature of the brain–lung interaction [84].

Collectively, these findings underscore the role of the glymphatic–lymphatic axis not merely as a CNS clearance mechanism but as a critical mediator of neurogenic systemic inflammation [85]. Understanding how impaired glymphatic function contributes to peripheral organ injury, particularly in the lungs, opens the door for novel therapeutic interventions aimed at preserving or restoring this system post-SAH.

### 2.4. Therapeutic Perspectives: Targeting Glymphatic and Lymphatic Pathways

**Figure 3:**
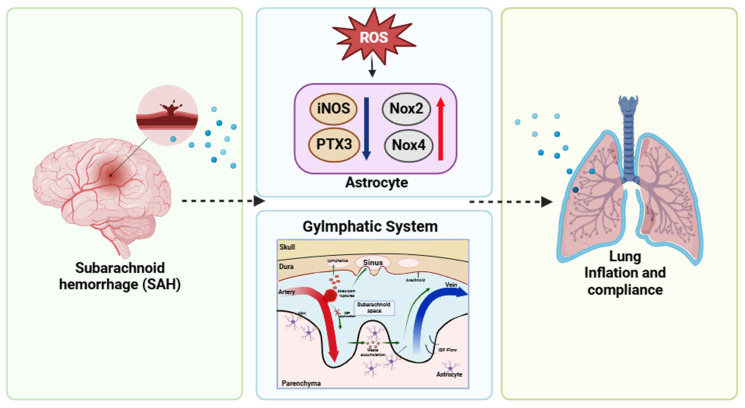
Mechanistic link between subarachnoid hemorrhage (SAH), glymphatic dysfunction, and pulmonary impairment. Subarachnoid hemorrhage (SAH) initiates a cascade involving astrocytic activation and increased production of reactive oxygen species (ROS), which upregulates iNOS, PTX3, Nox2, and Nox4. These molecular responses impair glymphatic function by disrupting cerebrospinal fluid (CSF) circulation. The resulting dysfunction may contribute to altered lung mechanics—specifically reduced inflation and compliance—via neuroimmune or humoral pathways. The diagram depicts a potential brain–lung interaction underlying systemic complications following SAH.

Subarachnoid hemorrhage (SAH) triggers a complex cascade of molecular and cellular responses that contribute to both brain and systemic dysfunctions. One of the key pathological features following SAH is the activation of astrocytes, which leads to the increased production of reactive oxygen species (ROS) [86]. These ROS subsequently upregulate several pro-inflammatory molecules, including inducible nitric oxide synthase (iNOS), pentraxin 3 (PTX3), NADPH oxidases Nox2 and Nox4. The overproduction of these molecules exacerbates inflammation and oxidative stress within the central nervous system (CNS), resulting in significant disruption of cerebrospinal fluid (CSF) circulation. This disruption impairs glymphatic function, which is crucial for the clearance of metabolic waste and maintaining brain homeostasis. The resulting glymphatic dysfunction may not only contribute to neuroinflammation but also to systemic complications such as pulmonary impairment [86] (Figure 3).

Given the central role of glymphatic dysfunction in the progression of both central and peripheral complications following SAH, therapeutic strategies aimed at restoring or enhancing glymphatic flow represent a promising and relatively underexplored avenue. Interventions targeting this system may not only mitigate secondary brain injury but also attenuate systemic inflammatory responses and reduce the incidence of associated pulmonary complications [87]. As the disruption of CSF flow and neuroinflammation are key drivers of both brain dysfunction and pulmonary impairment, targeting the glymphatic system could offer a dual benefit in improving neurological and pulmonary outcomes [88].

Several therapeutic approaches are currently under investigation, including the use of pharmacological agents to reduce oxidative stress, enhance astrocytic function, and restore normal CSF circulation. Potential preclinical and clinical evidence supporting these interventions suggests that targeting glymphatic and lymphatic pathways could lead to improved outcomes following SAH, particularly in minimizing secondary complications such as impaired lung mechanics, neuroimmune responses, and overall systemic inflammation [89,90].

#### 2.4.1. Modulating Aquaporin-4 Function

Aquaporin-4 (AQP4) channels are critical regulators of glymphatic inflow, facilitating the convective movement of CSF into the brain parenchyma [66,91,92]. After SAH, reactive astrogliosis leads to AQP4 depolarization, disrupting the directional water flow necessary for effective solute transport [66,86]. Preclinical studies have shown that genetic deletion of AQP4 impairs glymphatic function, while pharmacological agents that stabilize perivascular AQP4 polarization can restore flow and improve clearance of neurotoxic proteins such as amyloid-β and tau [93,94].

- Case Study and Research Findings:

One of the most promising pharmacological agents under investigation is TGN-020, a monoclonal antibody designed to stabilize AQP4 on astrocytic end feet [95]. In rodent models of traumatic brain injury (TBI) and SAH, TGN-020 administration has shown a significant restoration of glymphatic function, leading to reduced neuroinflammation and better cognitive recovery [96]. Preclinical studies have shown that stabilizing AQP4 through genetic or pharmacological means can enhance glymphatic clearance and reduce neurotoxic protein accumulation following subarachnoid hemorrhage (SAH). However, translating this approach to clinical settings remains challenging due to concerns regarding the specificity and delivery of AQP4-targeted therapies [60,97].

- Clinical Implications and Challenges:

Despite its promise, clinical translation of AQP4-targeted therapy faces several challenges, including the risk of off-target effects in the peripheral tissues, where AQP4 is also expressed [98,99]. Furthermore, achieving the required dose and precise localization of AQP4-modulating drugs in the human brain poses a significant hurdle, which may be mitigated through the development of nanocarriers or localized drug delivery systems [100,101]. Ongoing studies are investigating the use of AQP4 stabilizers in TBI patients, with a focus on improving cognitive outcomes and mitigating secondary brain injury.

#### 2.4.2. Enhancing CSF Dynamics

Maintaining adequate CSF circulation is essential for effective glymphatic clearance. Several therapeutic strategies aim to modulate the forces that drive CSF flow, including respiratory and arterial pulsations [90]. Pharmacological agents such as acetazolamide (which reduces intracranial pressure by inhibiting carbonic anhydrase) and furosemide (a loop diuretic that decreases cerebral edema) have been used in the past to manipulate CSF production and dynamics, though with mixed results [102,103].

- Innovative Approaches to CSF Dynamics:

More promising approaches involve mechanical interventions that target the glymphatic driving forces, such as respiratory modulation and arterial pulsations [104]. One such intervention is high-frequency oscillatory ventilation (HFOV), which has been shown to enhance CSF flow in preclinical models by increasing thoracic pressure oscillations. These oscillations not only support ventilation but also generate pressure gradients across the brain, thereby improving CSF influx and glymphatic clearance [105,106].

Another exciting area of research involves positive airway pressure (PAP) therapy. In clinical settings, PAP devices (such as continuous positive airway pressure, or Continuous positive airway pressure (CPAP) have been used to treat sleep apnea, but they may also have beneficial effects on glymphatic flow [107,108]. Studies in animal models have demonstrated that PAP improves CSF dynamics by promoting synchronized respiratory and circulatory activity, which is critical for glymphatic function. These findings suggest that respiratory-based therapies may have dual benefits for both pulmonary and central nervous system health in SAH patients [109].

- Sleep Enhancement and Glymphatic Activity:

Promoting slow-wave sleep (SWS) is another potential approach to enhancing glymphatic activity [110]. SWS has been shown to be a key phase during which the glymphatic system operates most efficiently, facilitating the clearance of toxic metabolites from the brain. Techniques that promote or prolong SWS, such as auditory stimulation or pharmacological agents that enhance sleep quality, may improve glymphatic function in SAH patients [111]. Experimental studies using melatonin and sleep-promoting agents (e.g., zolpidem) have shown potential benefits in increasing glymphatic clearance in animal models, though clinical trials are needed to confirm their efficacy in human patients [112].

#### 2.4.3. Targeting Meningeal Lymphatic Drainage

Recent discoveries have highlighted the significant role of meningeal lymphatic vessels in brain waste clearance, a function that is integral to maintaining glymphatic flow [24]. These vessels provide the primary efflux route, draining interstitial fluid and waste products such as neurotoxic proteins and DAMPs from the brain to the cervical lymphatic system [81,113]. This clearance is especially crucial following events such as subarachnoid hemorrhage (SAH), where disrupted lymphatic function can exacerbate neuroinflammation and impede the resolution of brain injury.

- Restoring Meningeal Lymphatic Function:

Restoring the function of meningeal lymphatic vessels is emerging as a promising therapeutic approach for improving the drainage of waste products and reducing secondary neuroinflammation. One of the most notable strategies is the use of VEGF-C (vascular endothelial growth factor C), a potent lymphangiogenic factor known to promote the formation and function of lymphatic vessels. Preclinical studies have shown that VEGF-C administration can enhance the growth and patency of meningeal lymphatic vessels, facilitating the clearance of neurotoxic substances from the brain [114,115,116]. This process not only improves waste removal but also enhances immune cell trafficking, further reducing neuroinflammation. Moreover, VEGF-C therapy has the potential to alleviate systemic immune activation by promoting the clearance of DAMPs from the brain [117,118]. This, in turn, could mitigate the development of secondary pulmonary complications, such as neurogenic pulmonary edema (NPE) and acute lung injury (ALI), which are common in the aftermath of SAH.

- Clinical Challenges and Potential:

Despite the promise of VEGF-C as a therapeutic strategy, several challenges remain, particularly in the context of clinical application [119]. One major concern is the delivery method of VEGF-C, as systemic administration could lead to unwanted lymph angiogenesis in peripheral tissues, potentially causing adverse effects [120,121]. To address this, researchers are exploring targeted delivery methods, such as intrathecal injection or intranasal administration, to localize the therapy to the brain and minimize systemic side effects. Another challenge is the long-term sustainability of VEGF-C therapy. Given that VEGF-C administration may promote lymph angiogenesis only transiently, efforts are being made to explore genetic modulation as a means of enhancing endogenous lymphatic function [122,123]. This approach could offer a more durable and effective solution for restoring meningeal lymphatic function, potentially leading to long-term benefits in brain health following SAH.

#### 2.4.4. Anti-Inflammatory and Neuroimmune Modulation

The close relationship between glymphatic dysfunction and neuroinflammation makes anti-inflammatory therapies an attractive avenue for therapeutic intervention. Pro-inflammatory cytokines such as TNF-α, IL-1β, and IL-6 have been shown to be elevated following SAH and play a crucial role in the secondary injury cascades [39,124]. Therefore, modulating neuroinflammation could indirectly improve glymphatic flow by preserving the integrity of perivascular structures and restoring normal vascular compliance.

- Cytokine Inhibition in SAH Models:

In preclinical studies, IL-1β antagonists and TNF-α inhibitors have been shown to reduce neuroinflammation and prevent AQP4 depolarization in animal models of SAH. Administration of an IL-1β antagonist within the first 24 h following subarachnoid hemorrhage (SAH) has been shown to improve glymphatic flow and result in better cognitive outcomes [125]. These results suggest that targeting specific cytokines during the acute phase of SAH could preserve glymphatic function and reduce the risk of neurogenic pulmonary complications.

- Emerging Anti-inflammatory Agents:

Agents targeting other inflammatory pathways, such as NLRP3 inflammasome inhibitors or oxidative stress modulators (e.g., N-acetylcysteine), could also prove beneficial in mitigating both neuroinflammation and glymphatic dysfunction [126]. The use of microglial modulators to limit neuroinflammation is another promising area of research.

#### 2.4.5. Emerging Technologies and Biomarker Development

Recent advances in imaging technologies have enabled real-time visualization and quantification of glymphatic function in vivo. Techniques such as contrast-enhanced MRI, two-photon microscopy, and transcranial ultrasound modulation allow researchers to monitor glymphatic flow and identify early signs of dysfunction in animal models. These technologies have significant potential for guiding clinical interventions in real-time, offering a personalized approach to SAH treatment [127].

- Biomarkers for Glymphatic Dysfunction:

In addition to imaging techniques, CSF biomarkers associated with glymphatic function, such as amyloid-β, tau, and lactate, can provide valuable insights into the functional status of the glymphatic system [128]. The development of non-invasive biomarkers to assess glymphatic clearance in SAH patients would enable more precise monitoring of therapeutic outcomes and provide early indicators of treatment efficacy.

## 3. Discussion

This review has explored the intricate pathophysiological relationship between subarachnoid hemorrhage (SAH), glymphatic dysfunction, and secondary pulmonary complications [44,129]. The evidence presented highlights the emerging understanding that the glymphatic–lymphatic system functions as more than a passive waste clearance mechanism. It is a dynamic and immunologically active interface that links the central nervous system (CNS) with peripheral organs, particularly the lungs, underscoring the importance of inter-organ communication in response to brain injury [48,104].

- Pathophysiological Mechanisms of Glymphatic Dysfunction and Systemic Inflammation:

SAH initiates a cascade of deleterious events that disrupt normal glymphatic flow. These events include abrupt increases in intracranial pressure, perivascular space obstruction by blood products, and reactive astrogliosis, which lead to AQP4 depolarization [66,91,92,98]. As a result, cerebrospinal fluid (CSF)-mediated solute exchange and waste clearance are impaired. This creates a toxic neurochemical environment, marked by sustained neuroinflammation, which is critical in exacerbating the secondary injury that occurs post-SAH [11]. Importantly, this CNS-localized inflammation is not restricted to the brain. It extends systemically through meningeal lymphatic pathways and a compromised blood–brain barrier (BBB), allowing damage-associated molecular patterns [56,130].

- Impact on Pulmonary Function and the Brain–Lung Axis:

Although AQP4 modulation primarily influences CNS fluid dynamics, its potential systemic effects are likely mediated through secondary mechanisms such as the release of pro-inflammatory cytokines and DAMPs into circulation. Clinically, aneurysmal subarachnoid hemorrhage (aSAH) can present with both neurogenic pulmonary edema (NPE) and Takotsubo cardiomyopathy (TCM) concurrently—an uncommon but well-documented phenomenon [131,132]. In a retrospective cohort, 7% of SAH patients developed both NPE and TCM, particularly in those with poor-grade hemorrhage and posterior circulation aneurysm [131]. This raises the possibility that glymphatic dysfunction may also contribute to a brain–heart axis, warranting further investigation. The lungs are particularly vulnerable to these circulating inflammatory signals. Studies in both humans and animal models consistently demonstrate that SAH frequently leads to neurogenic pulmonary edema and other forms of acute lung injury. These pulmonary complications not only worsen clinical outcomes but also have the potential to exacerbate CNS injury by promoting systemic hypoxia, inflammation, and impaired autoregulation. This creates a vicious cycle in which brain injury and lung dysfunction reinforce each other, significantly complicating patient management and recovery [18,133,134].

- Therapeutic Implications and Future Directions:

One of the most compelling aspects of this review is the potential for novel therapeutic interventions that target both glymphatic and lymphatic pathways to preserve neurological and pulmonary function. Strategies such as restoring AQP4 polarization, promoting meningeal lymph angiogenesis, or modulating neuroimmune signaling are particularly promising [113]. These approaches could help to re-establish normal waste clearance in the brain, reduce neuroinflammation, and improve lung function [135]. However, these interventions are still in the experimental phase, and their translation into clinical practice will require rigorous preclinical and clinical trials to assess their safety and efficacy. The complexity of the glymphatic–lymphatic system, combined with the need for targeted delivery methods, makes clinical implementation a challenging task. Despite this, these strategies represent a new frontier in the management of SAH and acute brain injury [24,38,50].

- Limitations and Knowledge Gaps:

Despite significant advances, several limitations remain in our understanding of the glymphatic system. The precise timing of glymphatic failure after SAH, and its relationship to clinical severity and long-term outcomes, has not been well-characterized. Additionally, interspecies differences in glymphatic anatomy and CSF dynamics complicate the extrapolation of findings from animal models to human physiology. Furthermore, there is a notable lack of clinically validated biomarkers or imaging tools to assess glymphatic function in real time [44,136]. This gap hinders our ability to diagnose glymphatic dysfunction early and monitor therapeutic interventions effectively. Developing reliable biomarkers and advanced imaging techniques will be critical for translating preclinical findings into clinical applications.

- Concluding Remarks: Toward Brain–Body Communication in Neurocritical Care:

Despite these challenges, the conceptual framework of a brain–body clearance and communication network offers exciting possibilities in neurocritical care and systems neuroscience. The glymphatic–lymphatic axis represents a new paradigm for understanding the interconnectedness of the brain and peripheral organs, emphasizing the need for a holistic approach to brain injury [18,24]. Future research should not only focus on restoring CNS homeostasis but also on addressing the systemic consequences of brain injury. A deeper understanding of the glymphatic–lymphatic system could ultimately provide new avenues for therapeutic intervention, breaking the cycle of neuroinflammation and peripheral organ dysfunction. By improving the clearance of waste products from the brain and reducing systemic inflammation, these strategies hold the potential to significantly enhance patient outcomes in SAH and other forms of acute brain injury [40,133,137].

## 4. Conclusions

The therapeutic landscape for addressing lymphatic dysfunction in subarachnoid hemorrhage is still in its infancy, but significant advances in our understanding of the lymphatic system and lymphoid tissue have led to promising therapeutic approaches. By targeting AQP4 function, improving CSF dynamics, restoring meningeal lymphatic function, and modulating neuroinflammation, both neurological and systemic complications after SAH appear to be mitigated. In addition, new technologies and biomarker development offer the potential for more precise and personalized treatment approaches. Future studies should focus on (1) developing reliable imaging and biomarker tools for real-time glymphatic assessment in SAH patients, (2) investigating early predictors of pulmonary complications via glymphatic–lymphatic dysfunction markers, (3) translating promising preclinical interventions such as AQP4 modulation or VEGF-C therapy to clinical trials, and (4) exploring integrated neuro–pulmonary critical care strategies to break the cycle of brain–lung injury.

## Data Availability

No new data were created or analyzed in this study.
